# Supplementary material for: Hydrogens and hydrogen-bond networks in macromolecular MicroED data
Source: J Struct Biol X. 2022 Nov 10;6:100078. doi: 10.1016/j.yjsbx.2022.100078 (PMC9731847; doi:10.1016/j.yjsbx.2022.100078)
Supplement: Supplementary data 1 [file mmc1.docx]

Supplementary Information

**Hydrogens and hydrogen-bond networks in macromolecular MicroED data**

Max T.B. Clabbers^1^, Michael W. Martynowycz^1,2^, Johan Hattne^1,2^, Tamir Gonen^1,2,3,*^

^1^Department of Biological Chemistry, University of California, Los Angeles CA 90095

^2^Howard Hughes Medical Institute, University of California, Los Angeles CA 90095

^3^Department of Physiology, University of California, Los Angeles CA 90095

*Corresponding author. E-mail address: [tgonen@g.ucla.edu](mailto:tgonen@g.ucla.edu)

**
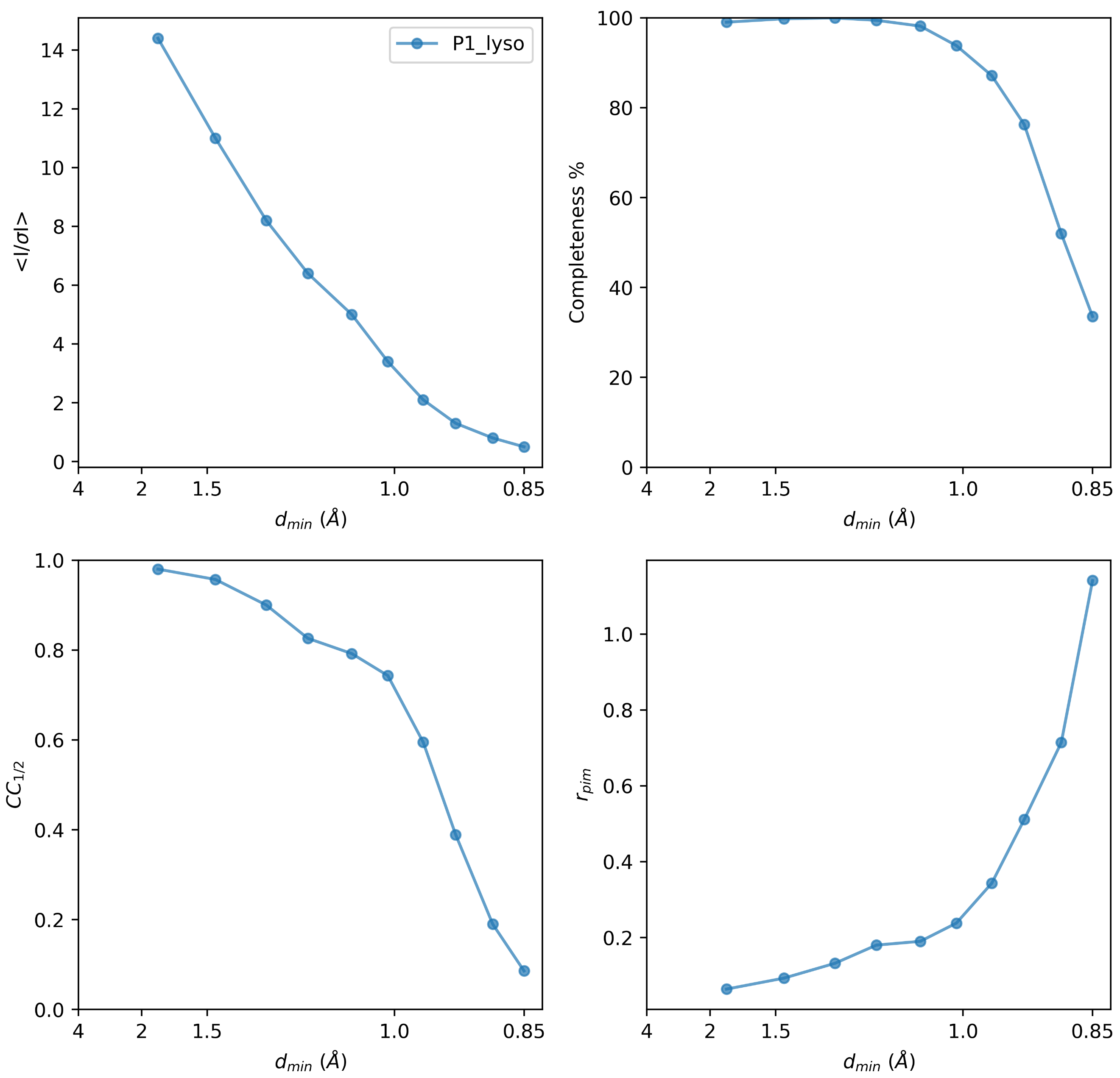
**

**Supplementary Figure 1.** Merging statistics for triclinic lysozyme. Crystallographic quality indicators and data completeness plotted as function of resolution for triclinic lysozyme at 0.87 Å resolution (see Supplementary Table 1).

**Supplementary Table 1.** MicroED data collection and refinement statistics

| **Data collection^*^** |  |
| --- | --- |
| Wavelength | 0.0197 |
| No. of crystals | 16 |
| Space group | *P*1 |
| Cell dimensions |  |
| *a, b, c* (Å) | 26.42, 30.72, 33.01 |
| *α, β, γ* (°) | 88.32, 109.10, 112.08 |
| Resolution (Å) | 16.05-0.87 (0.90-0.87)^**^ |
| Observed reflections | 569407 (5797) |
| Unique reflections | 64986 (2783) |
| Multiplicity | 8.8 (2.1) |
| Completeness (%) | 87.55 (37.64) |
| R_merge_ | 0.236 (1.035) |
| R_meas_ | 0.248 (1.409) |
| R_pim_ | 0.073 (0.945) |
| Mean I/σ(I) | 6.23 (0.66) |
| CC_1/2_ | 0.990 (0.147) |
| **Refinement** |  |
| No. of reflections | 64974 |
| No. of reflections used for R_free_ | 3168 |
| R_work_ / R_free_ | 0.197 / 0.220 |
| No. of atoms | 1216 |
| Proteins | 1081 |
| Ligand | 16 |
| Water | 119 |
| R.m.s. deviations |  |
| Bond lengths (Å) | 0.034 |
| Bond angles (°) | 2.447 |
| Mean *B*-factor (Å^2^) | 11.98 |
| Ramachandran |  |
| Favored (%) | 96.19 |
| Allowed (%) | 3.81 |
| Outliers (%) | 0.00 |
| Rotamer outliers (%) | 2.33 |

^*^Data from Martynowycz *et al.* 2022.

^**^Values in parentheses are for the highest-resolution shell.

**Supplementary Table 2.** Bond distances and angles for hydrogen bonding interactions

| Donor-H•••Acceptor | Diff. peak σ | D-H (Å) | H•••A (Å) | D•••A (Å) | D-H•••A (°) |
| --- | --- | --- | --- | --- | --- |
| Phe^3^-N-H•••Phe^38^-O | 2.50 | 1.18 | 1.67 | 2.81 | 160.72 |
| Ala^11^-N-H•••Glu^7^-O | 2.58 | 0.78 | 2.40 | 2.89 | 122.19 |
| Met^12^-N-H•••Leu^8^-O | 2.44 | 0.90 | 1.87 | 2.70 | 153.21 |
| Lys^13^-N-H•••Ala^9^-O | 3.44 | 1.21 | 1.59 | 2.76 | 162.32 |
| Arg^14^-N-H•••Ala^10^-O | 2.39 | 1.08 | 2.02 | 2.82 | 128.88 |
| His^15^-NE2-H•••Thr^89^-OG1 | 2.39 | 1.27 | 1.33 | 2.59 | 162.48 |
| Leu^17^-N-H•••Met^12^-O | 2.91 | 1.11 | 1.92 | 2.99 | 162.18 |
| Tyr^20^-N-H•••Leu^17^-O | 2.78 | 1.06 | 1.98 | 2.92 | 146.41 |
| Val^29^-N-H•••Leu^25^-O | 2.09 | 0.77 | 2.39 | 2.94 | 129.35 |
| Cys^30^-N-H•••Gly^26^-O | 2.45 | 1.00 | 1.85 | 2.74 | 146.82 |
| Ala^31^-N-H•••Asn^27^-O | 3.23 | 1.10 | 1.87 | 2.79 | 138.11 |
| Lys^33^-N-H•••Val^29^-O | 3.03 | 0.86 | 2.01 | 2.81 | 153.99 |
| Phe^34^-N-H•••Cys^30^-O | 3.21 | 0.79 | 2.18 | 2.95 | 169.63 |
| Thr^40^-N-H•••Lys^1^-O | 3.03 | 1.08 | 1.77 | 2.75 | 147.63 |
| Ala^42^-N-H•••Asn^39^-O | 3.27 | 1.20 | 1.77 | 2.89 | 154.32 |
| Asn^44^-N-H•••Asp^52^-O | 3.12 | 1.41 | 1.46 | 2.86 | 170.74 |
| Asn^46^-N-H•••Ser^50^-O | 2.56 | 1.21 | 1.63 | 2.73 | 148.76 |
| Asp^52^-N-H•••Asn^44^-O | 4.33 | 0.90 | 1.93 | 2.75 | 149.88 |
| Tyr^53^-O-H•••Asp^66^-OD2 | 3.44 | 1.39 | 1.25 | 2.59 | 157.86 |
| Gly^54^-N-H•••Thr^43^-O | 4.48 | 1.10 | 1.59 | 2.67 | 168.44 |
| Gln^57^-N-H•••Gly^54^-O | 3.01 | 1.11 | 1.83 | 2.84 | 150.17 |
| Asn^65^-N-H•••Leu^78^-O | 2.74 | 0.91 | 1.87 | 2.77 | 159.36 |
| Gly^67^-N-H•••Asn^65^-O | 3.90 | 1.13 | 1.85 | 2.90 | 152.42 |
| Arg^73^-N-H•••Arg^61^-O | 3.77 | 1.17 | 1.74 | 2.80 | 147.11 |
| Leu^75^-N-H•••Trp^62^-O | 2.86 | 0.84 | 1.93 | 2.75 | 161.86 |
| Ser^81^-N-H•••NO3^201^-O | 2.92 | 1.07 | 1.8 | 2.76 | 146.94 |
| Ala^82^-N-H•••Pro^70^-O | 2.16 | 1.18 | 1.71 | 2.79 | 148.60 |
| Leu^83^-N-H•••Cys^80^-O | 2.32 | 1.04 | 1.77 | 2.73 | 150.33 |
| Leu^84^-N-H•••Ser^81^-O | 2.99 | 1.03 | 1.92 | 2.93 | 164.49 |
| Val^92^-N-H•••Ile^88^-O | 3.00 | 0.98 | 1.79 | 2.77 | 174.55 |
| Asn^93^-N-H•••Thr^89^-O | 3.67 | 0.96 | 1.79 | 2.74 | 168.89 |
| Ala^95^-N-H•••Ser^91^-O | 2.74 | 0.87 | 1.86 | 2.73 | 172.72 |
| Lys^96^-N-H•••Val^92^-O | 4.21 | 1.05 | 1.76 | 2.81 | 174.10 |
| Ile^98^-N-H•••Cys^94^-O | 3.04 | 1.13 | 1.72 | 3.15 | 152.39 |
| Val^99^-N-H•••Ala^95^-O | 3.06 | 1.09 | 1.95 | 3.03 | 171.83 |
| Trp^108^-N-H•••Met^105^-O | 3.10 | 1.26 | 1.64 | 2.82 | 152.36 |
| Trp^111^-NE1-H•••Asn^27^-OD1 | 3.39 | 1.02 | 1.79 | 2.71 | 169.48 |
| Arg^114^-N-H•••Arg^110^-O | 2.27 | 1.11 | 1.98 | 2.76 | 124.52 |
| Trp^123^-N-H•••Val^120^-O | 2.45 | 1.04 | 1.83 | 2.84 | 161.33 |

**Supplementary Table 3.** Hydrogen bond distances for Cα-H

| Residue | Name | Atom | Diff. peak σ | X-H (Å) |
| --- | --- | --- | --- | --- |
| 2 | Val | CA | 3.42 | 1.15 |
| 3 | Phe | CA | 3.46 | 1.26 |
| 4 | Gly | CA | 3.18 | 1.14 |
| 4 | Gly | CA | 2.39 | 0.98 |
| 5 | Arg | CA | 2.59 | 1.27 |
| 7 | Glu | CA | 4.16 | 1.06 |
| 9 | Ala | CA | 3.79 | 1.08 |
| 10 | Ala | CA | 2.74 | 1.00 |
| 11 | Ala | CA | 2.78 | 0.81 |
| 12 | Met | CA | 2.74 | 1.11 |
| 13 | Lys | CA | 2.01 | 1.20 |
| 16 | Gly | CA | 2.60 | 0.89 |
| 18 | Asp | CA | 2.26 | 1.45 |
| 19 | Asn | CA | 2.81 | 1.43 |
| 20 | Tyr | CA | 3.17 | 1.10 |
| 21 | Arg | CA | 3.12 | 1.29 |
| 22 | Gly | CA | 2.77 | 1.18 |
| 24 | Ser | CA | 3.96 | 1.10 |
| 25 | Leu | CA | 3.06 | 1.19 |
| 27 | Asn | CA | 3.22 | 1.16 |
| 28 | Trp | CA | 3.34 | 1.10 |
| 29 | Val | CA | 2.42 | 1.10 |
| 32 | Ala | CA | 2.39 | 1.05 |
| 33 | Lys | CA | 2.71 | 1.05 |
| 38 | Phe | CA | 2.41 | 1.18 |
| 39 | Asn | CA | 2.96 | 0.99 |
| 42 | Ala | CA | 2.17 | 0.83 |
| 44 | Asn | CA | 2.16 | 0.90 |
| 45 | Arg | CA | 2.81 | 0.98 |
| 51 | Thr | CA | 3.55 | 0.90 |
| 52 | Asp | CA | 3.04 | 1.20 |
| 53 | Tyr | CA | 2.27 | 1.14 |
| 54 | Gly | CA | 3.43 | 1.17 |
| 55 | Ile | CA | 2.63 | 1.12 |
| 56 | Ile | CA | 2.85 | 1.25 |
| 57 | Asn | CA | 2.89 | 1.23 |
| 58 | Ile | CA | 3.49 | 0.97 |
| 59 | Asn | CA | 3.51 | 1.06 |
| 62 | Trp | CA | 2.78 | 1.18 |
| 63 | Trp | CA | 2.29 | 1.25 |
| 69 | Thr | CA | 2.61 | 1.00 |
| 70 | Pro | CA | 2.91 | 1.06 |
| 74 | Asn | CA | 3.22 | 1.03 |
| 75 | Leu | CA | 2.65 | 1.13 |
| 76 | Cys | CA | 2.76 | 1.08 |
| 77 | Asn | CA | 2.87 | 1.13 |
| 82 | Ala | CA | 3.27 | 1.09 |
| 88 | Ile | CA | 2.64 | 1.41 |
| 91 | Ser | CA | 4.18 | 1.40 |
| 94 | Cys | CA | 2.89 | 1.02 |
| 96 | Lys | CA | 2.80 | 1.05 |
| 97 | Lys | CA | 2.38 | 1.24 |
| 99 | Val | CA | 3.06 | 1.15 |
| 103 | Asn | CA | 2.14 | 1.17 |
| 108 | Trp | CA | 4.03 | 0.94 |
| 110 | Ala | CA | 3.41 | 1.06 |
| 111 | Trp | CA | 2.44 | 1.10 |
| 116 | Lys | CA | 2.93 | 1.11 |
| 117 | Gly | CA | 4.02 | 1.11 |
| 119 | Asp | CA | 3.19 | 1.07 |
| 123 | Trp | CA | 2.60 | 1.14 |

**Supplementary Table 4.** Hydrogen bond distances for side chain C-H

| Residue | Name | Atom | Diff. peak σ | X-H (Å) |
| --- | --- | --- | --- | --- |
| 8 | Leu | CG | 2.50 | 0.94 |
| 15 | His | CE1 | 2.50 | 1.45 |
| 40 | Thr | CB | 2.64 | 1.51 |
| 56 | Leu | CG | 3.74 | 1.22 |
| 58 | Ile | CB | 3.35 | 1.18 |
| 63 | Trp | CD1 | 3.06 | 1.39 |
| 69 | Thr | CB | 3.08 | 0.99 |
| 84 | Leu | CG | 2.88 | 1.29 |
| 89 | Thr | CB | 3.24 | 1.25 |
| 98 | Ile | CB | 2.19 | 1.06 |
| 108 | Trp | CD1 | 2.84 | 1.25 |
| 109 | Val | CB | 2.36 | 1.51 |
| 118 | Thr | CB | 3.39 | 1.29 |
| 123 | Trp | CD1 | 2.44 | 1.19 |

**Supplementary Table 5.** Hydrogen bond distances for aromatic C-H

| Residue | Name | Atom | Diff. peak σ | X-H (Å) |
| --- | --- | --- | --- | --- |
| 3 | Phe | CE2 | 3.12 | 1.05 |
| 20 | Tyr | CE1 | 3.09 | 0.99 |
| 23 | Tyr | CD2 | 4.18 | 1.01 |
| 23 | Tyr | CD1 | 2.81 | 1.11 |
| 23 | Tyr | CE1 | 2.15 | 1.32 |
| 38 | Phe | CZ | 3.97 | 1.13 |
| 38 | Phe | CE2 | 2.56 | 1.32 |
| 38 | Phe | CD1 | 2.11 | 1.03 |
| 53 | Tyr | CD1 | 3.52 | 1.18 |
| 53 | Tyr | CE2 | 3.39 | 1.16 |
| 63 | Trp | CZ3 | 3.51 | 1.00 |
| 63 | Trp | CE3 | 3.19 | 1.22 |
| 108 | Trp | CE3 | 3.86 | 1.07 |
| 108 | Trp | CH2 | 3.47 | 1.01 |
| 108 | Trp | CZ3 | 2.42 | 1.31 |
| 111 | Trp | CH2 | 3.52 | 1.24 |
| 111 | Trp | CZ2 | 2.30 | 1.02 |

**Supplementary Table 6.** Hydrogen bond distances for CH_2_

| Residue | Name | Atom | Diff. peak σ | X-H (Å) |
| --- | --- | --- | --- | --- |
| 1 | Lys | CE | 3.20 | 1.41 |
| 1 | Lys | CE | 2.30 | 1.17 |
| 1 | Lys | CG | 3.15 | 1.05 |
| 1 | Lys | CB | 3.12 | 1.16 |
| 3 | Phe | CB | 3.37 | 0.98 |
| 3 | Phe | CB | 2.43 | 1.14 |
| 5 | Arg | CB | 3.12 | 1.23 |
| 5 | Arg | CB | 2.81 | 1.46 |
| 5 | Arg | CG | 2.20 | 1.14 |
| 6 | Cys | CB | 2.37 | 1.04 |
| 7 | Glu | CB | 2.42 | 1.58 |
| 8 | Leu | CB | 2.45 | 1.03 |
| 8 | Leu | CB | 2.43 | 1.10 |
| 12 | Met | CG | 3.12 | 1.18 |
| 14 | Arg | CG | 3.82 | 1.00 |
| 15 | His | CB | 2.07 | 1.16 |
| 18 | Asp | CB | 2.84 | 1.00 |
| 18 | Asp | CB | 2.29 | 1.21 |
| 19 | Asn | CB | 3.04 | 1.01 |
| 19 | Asn | CB | 2.48 | 1.06 |
| 20 | Tyr | CB | 2.43 | 1.06 |
| 20 | Tyr | CB | 2.20 | 1.07 |
| 21 | Arg | CG | 3.36 | 1.17 |
| 21 | Arg | CB | 2.38 | 1.16 |
| 21 | Arg | CB | 2.16 | 1.47 |
| 23 | Trp | CB | 2.80 | 1.14 |
| 25 | Leu | CB | 3.22 | 1.08 |
| 27 | Asn | CB | 2.69 | 0.91 |
| 28 | Trp | CB | 2.19 | 1.06 |
| 33 | Lys | CE | 3.45 | 1.07 |
| 33 | Lys | CG | 3.11 | 1.08 |
| 33 | Lys | CG | 2.27 | 1.13 |
| 34 | Phe | CB | 4.37 | 1.18 |
| 35 | Glu | CG | 3.11 | 1.17 |
| 35 | Glu | CG | 2.53 | 1.34 |
| 36 | Ser | CB | 3.51 | 1.09 |
| 36 | Ser | CB | 2.63 | 1.51 |
| 38 | Phe | CB | 3.14 | 1.06 |
| 41 | Gln | CB | 3.12 | 1.19 |
| 41 | Gln | CB | 2.76 | 1.00 |
| 41 | Gln | CG | 2.48 | 1.10 |
| 48 | Asp | CB | 2.11 | 1.29 |
| 57 | Gln | CB | 3.45 | 1.18 |
| 57 | Gln | CB | 2.49 | 1.19 |
| 58 | Ile | CG1 | 3.79 | 1.23 |
| 58 | Ile | CG1 | 2.79 | 1.12 |
| 59 | Asn | CB | 3.11 | 1.15 |
| 59 | Asn | CB | 2.52 | 1.06 |
| 60 | Ser | CB | 3.67 | 1.30 |
| 61 | Arg | CB | 4.18 | 1.04 |
| 61 | Arg | CB | 2.59 | 0.91 |
| 61 | Arg | CG | 2.40 | 1.47 |
| 62 | Trp | CB | 3.11 | 1.25 |
| 63 | Trp | CB | 2.97 | 1.29 |
| 63 | Trp | CB | 2.34 | 1.29 |
| 64 | Cys | CB | 2.55 | 1.07 |
| 66 | Asp | CB | 2.69 | 1.19 |
| 66 | Asp | CB | 2.26 | 1.43 |
| 68 | Arg | CB | 4.31 | 1.16 |
| 70 | Pro | CB | 2.76 | 1.27 |
| 72 | Ser | CB | 2.04 | 1.14 |
| 73 | Arg | CB | 2.22 | 1.18 |
| 74 | Asn | CB | 2.46 | 1.16 |
| 74 | Asn | CB | 2.14 | 1.27 |
| 75 | Leu | CB | 2.82 | 1.35 |
| 76 | Cys | CB | 3.12 | 0.76 |
| 76 | Cys | CB | 3.07 | 1.21 |
| 77 | Asn | CB | 2.76 | 1.08 |
| 78 | Ile | CG1 | 2.13 | 1.05 |
| 80 | Cys | CB | 3.14 | 0.99 |
| 80 | Cys | CB | 2.60 | 1.31 |
| 83 | Leu | CB | 2.57 | 1.01 |
| 84 | Leu | CB | 2.72 | 1.23 |
| 85 | Ser | CB | 2.37 | 1.28 |
| 86 | Ser | CB | 2.91 | 1.22 |
| 87 | Asp | CB | 3.81 | 1.20 |
| 88 | Ile | CG1 | 2.16 | 1.34 |
| 91 | Ser | CB | 2.86 | 0.95 |
| 91 | Ser | CB | 2.10 | 1.16 |
| 93 | Asn | CB | 3.61 | 1.40 |
| 93 | Asn | CB | 2.59 | 1.15 |
| 94 | Cys | CB | 3.46 | 1.08 |
| 94 | Cys | CB | 2.50 | 1.20 |
| 96 | Lys | CB | 3.05 | 0.90 |
| 97 | Lys | CD | 4.05 | 1.25 |
| 97 | Lys | CG | 3.62 | 1.07 |
| 97 | Lys | CG | 2.88 | 1.26 |
| 97 | Lys | CD2 | 2.97 | 1.30 |
| 97 | Lys | CD2 | 2.65 | 1.18 |
| 97 | Lys | CB | 2.60 | 0.82 |
| 100 | Ser | CB | 3.19 | 1.24 |
| 105 | Met | CB | 2.62 | 1.42 |
| 105 | Met | CG | 2.35 | 1.23 |
| 108 | Trp | CB | 3.41 | 1.17 |
| 114 | Arg | CB | 2.26 | 1.16 |
| 116 | Lys | CE | 3.61 | 1.23 |
| 121 | Gln | CB | 2.77 | 1.04 |
| 121 | Gln | CB | 2.50 | 1.23 |
| 123 | Trp | CB | 2.11 | 1.41 |

**Supplementary Table 7.** Hydrogen bond distances for CH_3_

| Residue | Name | Atom | Diff. peak σ | X-H (Å) |
| --- | --- | --- | --- | --- |
| 2 | Val | CG1 | 3.42 | 0.94 |
| 2 | Val | CG1 | 2.23 | 1.15 |
| 2 | Val | CG2 | 3.04 | 1.31 |
| 2 | Val | CG2 | 2.63 | 1.11 |
| 8 | Leu | CD1 | 3.59 | 1.20 |
| 8 | Leu | CD1 | 3.12 | 0.99 |
| 9 | Ala | CB | 2.93 | 1.00 |
| 10 | Ala | CB | 3.73 | 1.12 |
| 10 | Ala | CB | 2.89 | 1.54 |
| 10 | Ala | CB | 2.56 | 1.03 |
| 12 | Met | CE | 3.02 | 1.14 |
| 12 | Met | CE | 2.27 | 1.11 |
| 17 | Leu | CD2 | 2.75 | 0.99 |
| 17 | Leu | CD1 | 2.52 | 0.90 |
| 25 | Leu | CD2 | 3.61 | 0.87 |
| 25 | Leu | CD2 | 3.04 | 1.12 |
| 25 | Leu | CD1 | 2.31 | 1.03 |
| 29 | Val | CG1 | 3.91 | 0.99 |
| 29 | Val | CG1 | 2.92 | 1.40 |
| 29 | Val | CG2 | 2.42 | 1.06 |
| 31 | Ala | CB | 2.64 | 0.91 |
| 31 | Ala | CB | 2.47 | 1.05 |
| 31 | Ala | CB | 2.53 | 0.87 |
| 32 | Ala | CB | 2.63 | 0.90 |
| 40 | Thr | CG2 | 2.99 | 1.28 |
| 42 | Ala | CB | 2.71 | 0.94 |
| 51 | Thr | CG2 | 3.29 | 1.18 |
| 51 | Thr | CG2 | 2.31 | 1.12 |
| 55 | Ile | CD1 | 2.71 | 1.12 |
| 55 | Ile | CD1 | 2.30 | 1.20 |
| 55 | Ile | CG2 | 2.54 | 0.97 |
| 56 | Leu | CD1 | 3.20 | 1.35 |
| 56 | Leu | CD1 | 2.52 | 1.06 |
| 56 | Leu | CD1 | 2.56 | 1.31 |
| 56 | Leu | CD2 | 2.67 | 0.99 |
| 56 | Leu | CD2 | 2.47 | 1.14 |
| 58 | Ile | CD1 | 2.82 | 1.09 |
| 58 | Ile | CD1 | 2.54 | 1.09 |
| 58 | Ile | CG2 | 3.20 | 1.26 |
| 58 | Ile | CG2 | 3.00 | 0.87 |
| 69 | Thr | CG2 | 2.05 | 1.21 |
| 75 | Leu | CD1 | 3.45 | 1.08 |
| 75 | Leu | CD1 | 2.35 | 0.89 |
| 75 | Leu | CD2 | 2.27 | 0.95 |
| 78 | Ile | CG2 | 3.55 | 1.27 |
| 78 | Ile | CG2 | 2.34 | 1.25 |
| 82 | Ala | CB | 3.39 | 0.89 |
| 82 | Ala | CB | 3.12 | 1.04 |
| 83 | Leu | CD2 | 2.34 | 1.36 |
| 84 | Leu | [CD2](mailto:CD@-HD22) | 3.58 | 1.18 |
| 84 | Leu | [CD1](mailto:CD@-HD22) | 2.80 | 1.02 |
| 88 | Ile | CD1 | 3.97 | 1.27 |
| 88 | Ile | CD1 | 2.12 | 1.36 |
| 88 | Ile | CG2 | 2.43 | 1.09 |
| 88 | Ile | CG2 | 2.21 | 1.16 |
| 90 | Ala | CB | 4.32 | 1.13 |
| 92 | Val | CG2 | 3.67 | 0.92 |
| 92 | Val | CG2 | 3.34 | 0.81 |
| 92 | Val | CG2 | 3.08 | 1.02 |
| 95 | Ala | CB | 2.63 | 1.02 |
| 95 | Ala | CB | 2.19 | 0.96 |
| 98 | Ile | CD1 | 3.03 | 1.19 |
| 98 | Ile | CD1 | 2.15 | 0.79 |
| 99 | Val | CG2 | 2.93 | 1.13 |
| 99 | Val | CG2 | 2.79 | 1.07 |
| 105 | Met | CE | 3.46 | 1.16 |
| 107 | Ala | CB | 2.74 | 1.32 |
| 110 | Ala | CB | 2.43 | 1.39 |
| 110 | Ala | CB | 2.03 | 0.96 |
| 118 | Thr | CG2 | 2.09 | 1.03 |
| 120 | Val | CG2 | 3.01 | 1.08 |
| 120 | Val | CG2 | 2.09 | 0.76 |
| 120 | Val | CG1 | 2.42 | 1.59 |
| 122 | Ala | CB | 2.64 | 1.17 |
| 122 | Ala | CB | 2.03 | 1.14 |
| 124 | Ile | CG2 | 2.79 | 0.98 |
| 124 | Ile | CG2 | 2.67 | 0.65 |

**Supplementary Table 8.** Hydrogen bond distances for N-H

| Residue | Name | Atom | Diff. peak σ | X-H (Å) |
| --- | --- | --- | --- | --- |
| 4 | Gly | N | 3.10 | 0.94 |
| 6 | Cys | N | 2.34 | 1.24 |
| 7 | Glu | N | 2.72 | 0.87 |
| 14 | Arg | NE1 | 2.47 | 1.19 |
| 18 | Asp | N | 2.90 | 0.91 |
| 21 | Arg | N | 3.12 | 1.26 |
| 22 | Gly | N | 3.94 | 0.80 |
| 23 | Tyr | N | 4.43 | 1.13 |
| 25 | Leu | N | 2.62 | 0.93 |
| 26 | Gly | N | 3.30 | 1.00 |
| 37 | Asn | N | 2.66 | 1.09 |
| 38 | Phe | N | 2.98 | 1.18 |
| 39 | Asn | N | 3.20 | 1.14 |
| 45 | Arg | N | 2.35 | 0.75 |
| 49 | Gly | N | 2.70 | 0.98 |
| 55 | Ile | N | 2.70 | 0.88 |
| 56 | Ile | N | 2.72 | 1.21 |
| 58 | Ile | N | 3.27 | 1.08 |
| 60 | Ser | N | 3.37 | 0.91 |
| 61 | Arg | N | 4.43 | 0.94 |
| 62 | Trp | N | 2.96 | 0.81 |
| 62 | Trp | NE1 | 2.35 | 1.11 |
| 71 | Gly | N | 2.21 | 1.12 |
| 72 | Ser | N | 2.27 | 1.07 |
| 74 | Asn | N | 2.06 | 0.73 |
| 76 | Cys | N | 2.76 | 1.19 |
| 78 | Ile | N | 2.58 | 0.85 |
| 80 | Cys | N | 2.11 | 0.98 |
| 86 | Ser | N | 2.12 | 1.12 |
| 88 | Ile | N | 2.09 | 0.91 |
| 89 | Thr | N | 4.00 | 1.14 |
| 90 | Ala | N | 3.27 | 1.13 |
| 91 | Ser | N | 2.14 | 0.99 |
| 104 | Gly | N | 2.87 | 1.24 |
| 105 | Met | N | 2.64 | 1.25 |
| 107 | Ala | N | 3.15 | 0.71 |
| 108 | Trp | NE1 | 2.37 | 1.14 |
| 109 | Val | N | 3.68 | 0.81 |
| 111 | Trp | N | 3.22 | 1.21 |
| 118 | Thr | N | 4.54 | 1.02 |
| 119 | Asp | N | 2.50 | 1.26 |
| 120 | Val | N | 3.86 | 1.16 |
| 121 | Asn | N | 2.50 | 0.88 |
| 124 | Ile | N | 3.53 | 0.84 |

**Supplementary Table 9.** Hydrogen bond distances for N-H_2_

| Residue | Name | Atom | Diff. peak σ | X-H (Å) |
| --- | --- | --- | --- | --- |
| 5 | Arg | NH1 | 2.43 | 1.14 |
| 14 | Arg | NH2 | 2.96 | 0.76 |
| 14 | Arg | NH1 | 2.93 | 1.26 |
| 19 | Asn | ND2 | 2.63 | 1.31 |
| 19 | Asn | ND2 | 2.37 | 1.14 |
| 27 | Asn | ND2 | 3.07 | 0.87 |
| 27 | Asn | ND2 | 2.00 | 1.13 |
| 39 | Asn | ND2 | 2.18 | 1.17 |
| 57 | Gln | NE2 | 2.80 | 1.00 |
| 59 | Asn | ND2 | 2.21 | 0.75 |
| 62 | Arg | NH1 | 2.42 | 1.10 |
| 74 | Asn | ND2 | 3.40 | 1.50 |
| 114 | Arg | NH1 | 2.07 | 0.92 |

**Supplementary Table 10.** Hydrogen bond distances for N-H_3_

| Residue | Name | Atom | Diff. peak σ | X-H (Å) |
| --- | --- | --- | --- | --- |
| 1 | Lys | NZ | 3.32 | 1.24 |
| 33 | Lys | NZ | 2.70 | 1.13 |
| 97 | Lys | NZ | 2.96 | 1.02 |
